# Comparative Investigation of GnRH‐Immunocastration and a Phytogenic Feed Additive on Reproductive Parameters, Testicular Echogenicity and Behavioural Activity in Young Holstein Bulls

**DOI:** 10.1111/rda.70305

**Published:** 2026-08-13

**Authors:** Nurdan Coşkun, Oğuz Kaan Yalçin, Murat Yüksel, Cafer Tayyar Ateş, Hasan Basri Dingil, Fikret Karaca

**Affiliations:** ^1^ Faculty of Veterinary Medicine, Department of Reproduction and Artificial Insemination Hatay Mustafa Kemal University Hatay Türkiye; ^2^ Faculty of Veterinary Medicine, Department of Obstetrics and Gynecology Hatay Mustafa Kemal University Hatay Türkiye; ^3^ Faculty of Veterinary Medicine, Department of Animal Science Hatay Mustafa Kemal University Hatay Türkiye

**Keywords:** anti‐GnRH vaccination, behavioural activity, phytogenic feed additive, scrotal circumference, testicular echotexture, young bulls

## Abstract

Managing aggressive behaviour in bulls often involves a trade‐off between handling ease and the anabolic benefits associated with endogenous testosterone production. This study compared the effects of GnRH‐immunocastration and a phytogenic feed additive on reproductive parameters, testicular echotexture and behavioural activity in young Holstein bulls aged 12–15 months. Bulls were allocated to either an immunocastration group (IC, *n* = 6) or a phytogenic feed additive group (PFA, *n* = 7). The IC group received two subcutaneous injections of 1 mL Bopriva (400 μg GnRH‐protein conjugate) at a three‐week interval, whereas the PFA group received 50 g/day of a phytogenic feed additive throughout the 90‐day experimental period. Evaluations included scrotal circumference (SC), serum testosterone concentrations, ultrasonographic testicular echotexture assessed by mean grey value (MGV) and behavioural observations. Repeated measurements obtained throughout the study were analysed using linear mixed models. Significant group × time interactions (*p* < 0.001) were observed for SC, testosterone concentrations and MGV. In the IC group, testosterone concentrations and SC progressively decreased beginning on Day 15, while MGV values declined from Day 30 onward. In contrast, the PFA group maintained relatively stable testosterone concentrations and ultrasonographic echotexture throughout the observation period. Qualitative behavioural observations indicated reduced mounting attempts and restlessness in both groups during the follow‐up period. Immunocastration induced marked suppression of reproductive parameters and alterations in testicular echotexture in young bulls. Under the conditions of this study, supplementation with the phytogenic feed additive was associated with relatively stable reproductive parameters, while qualitative reductions in behavioural activity were also observed. Further studies including untreated control groups and quantitative behavioural scoring systems are warranted.

## 1 Introduction

Castration has long been a standard management practice in male animals employed to mitigate aggressive behaviour, prevent unintended reproduction and eliminate undesirable odours (Cook et al. 2000; Brunius et al. 2011; Garside et al. 2014). In the specific context of cattle production, this procedure is frequently practiced to suppress sexual and social dominance behaviours, thereby facilitating easier herd management and ensuring safety during transport (Romans et al. 1994; Goodrich and Stricklin 1997). However, beyond these management advantages, the welfare significance of the procedure remains a critical point of discussion (Stafford and Mellor 2005). Recent research has increasingly focused on the physiological and structural impacts of castration, exploring how different methods influence testicular morphology and endocrine function (Monleón et al. 2020; Curtis et al. 2022). Despite its widespread use, traditional surgical, mechanical and chemical methods remain increasingly scrutinized due to animal welfare concerns. Although anaesthetics and analgesics are frequently employed to mitigate procedural pain, these conventional techniques are still limited by the risks of acute stress and post‐operative infections (Coetzee et al. 2010; Coetzee 2013; Ede et al. 2022). Furthermore, castration‐related stress reduces feed and water intake, suppresses immune function and impairs growth performance in young bulls (Warnock et al. 2014; Marti et al. 2015). Consequently, developing more humane and effective alternatives is essential for enhancing animal well‐being and ensuring sustainable livestock production.

In this context, immunocastration has emerged as a safe, effective and reversible alternative by targeting the hypothalamic–pituitary–gonadal (HPG) axis (Ulker et al. 2009; Han et al. 2015). This approach is based on active immunization against gonadotropin‐releasing hormone (GnRH), which triggers the production of specific antibodies that subsequently inhibit the secretion of gonadotropins, thereby suppressing testosterone production and spermatogenesis (Adams et al. 1993; D'Occhio 1993). Recent studies have demonstrated that vaccination with Bopriva significantly decreases testicular development and serum testosterone concentrations, while diminishing physical activity in both prepubertal and pubertal bulls (Janett et al. 2012a, 2012b). Given that testosterone plays a central role in male sexual development and libido, its hormonal suppression serves as the primary mechanism for achieving the desired management effects without the physical trauma of surgery (Hafez et al. 2004). Active immunization against GnRH effectively reduces testosterone secretion, leading to inhibited testicular development and a significant decrease in aggressive behaviours (Zanella et al. 2009; Janett et al. 2012a, 2012b). However, a notable drawback of immunocastration is the simultaneous loss of the anabolic benefits of testosterone, which are essential for maximizing weight gain and improving feed efficiency in intensive finishing systems (Cook et al. 2000; D'Occhio et al. 2001; Amatayakul‐Chantler et al. 2012). This trade‐off between behavioural control and growth performance necessitates the exploration of alternative strategies that can modulate sexual behaviour while preserving the growth potential of young bulls. Whereas GnRH immunization successfully manages behavioural issues, the resulting decline in androgen concentrations can lead to a reduction in average daily gain (ADG) and a higher feed conversion ratio (FCR) compared to intact bulls (Janett et al. 2012a; Miguel et al. 2014). To address this limitation, non‐pharmacological phytogenic feed additives aimed at reducing stress and aggressive behaviour have gained attention. These products, often containing specific essential oil blends and herbal extracts, are designed to modulate the central nervous system (CNS) without suppressing the HPG axis, a mechanism recently explored for stress management in beef cattle (Davis et al. 2017; Mallette 2023). Such an approach suggests that sexual and social behaviours could be managed while potentially preserving the growth‐promoting effects of endogenous androgens. Such phytogenic strategies represent a novel approach to achieving behavioural sedation while maintaining relatively stable testosterone concentrations and ultrasonographic echotexture. In this study, we hypothesised that either GnRH immunocastration or administration of a calming phytogenic feed additive could effectively mitigate undesirable behaviours, such as mounting and aggression, in young bulls. Therefore, the objective was to compare the efficacy of anti‐GnRH vaccination and phytogenic feed additive on scrotal circumference, testicular echogenicity, serum testosterone concentrations and physical activity in young Holstein bulls over 90 days.

## 2 Materials and Methods

All experimental procedures involving animals were conducted in accordance with national and international guidelines for the care and use of animals in research. The study protocol was approved by the Hatay Mustafa Kemal University Local Animal Experiments Ethics Committee (Approval No: 2024/03‐06).

### 2.1 Animals and Experimental Design

The study was conducted on 13 clinically healthy Holstein bulls aged between 12 and 15 months (mean ± SD: 13.4 ± 1.1), housed in a private commercial cattle enterprise under field conditions. Before the study, all animals underwent a complete clinical and reproductive examination to confirm the absence of systemic disease or reproductive abnormalities. Bulls were managed under identical environmental and nutritional conditions throughout the experimental period and had ad libitum access to fresh drinking water. The animals had a mean initial body weight of 288.38 ± 26.34 kg (range: 257–334 kg) and a mean initial scrotal circumference of 27.38 ± 3.74 cm. All animal care and daily management procedures were carried out by the farm owner, an experienced practitioner, ensuring consistent handling and standardised husbandry throughout the experimental period. The relatively moderate body weights observed in the present study likely reflected the enterprise's forage‐based feeding strategy and field management conditions, in which wheat straw was the primary roughage source. The study was designed as a longitudinal comparative trial evaluating two non‐surgical management approaches. Due to animal management constraints and animal welfare considerations associated with maintaining untreated aggressive bulls under commercial conditions, an untreated control group was not included. Instead, pre‐treatment (Day 0) measurements were used as baseline reference values throughout the study, allowing each bull to serve as its own internal control. Longitudinal physiological and behavioural responses were evaluated using repeated‐measures analyses, with particular emphasis on the group × time interaction. This design and its inherent limitations were taken into account during the statistical analysis and interpretation of the results. Following the initial clinical examination, animals were randomly assigned to one of two treatment groups. Bulls were randomly allocated to the immunocastration (IC; *n* = 6) and phytogenic feed additive (PFA; *n* = 7) groups. Baseline evaluations, including clinical examination, behavioural assessments, scrotal circumference measurements, testicular ultrasonography and blood sampling for serum testosterone analysis, were completed immediately before initiation of treatments (Day 0). The same evaluations were subsequently repeated on Days 15, 30, 45, 60, 75 and 90.

**FIGURE 1:**
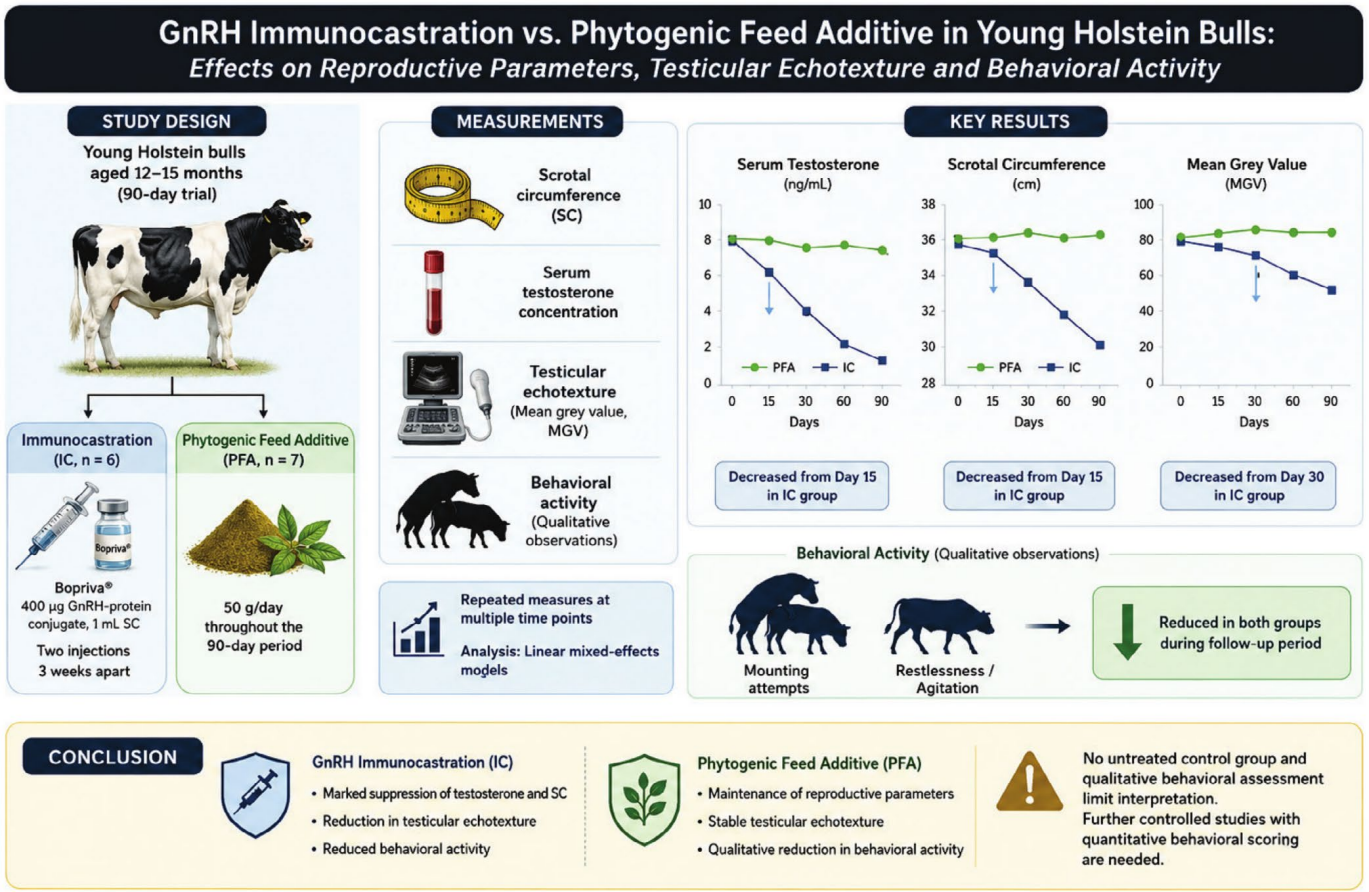
Schematic representation of the experimental design. Following baseline evaluations performed on Day 0, 13 Holstein bulls were randomly allocated to the immunocastration (IC; *n* = 6) or phytogenic feed additive (PFA; *n* = 7) group. Bulls in the IC group received Bopriva injections on Days 0 and 21, whereas animals in the PFA group received Stop‐Hop premix (50 g/head/day) mixed with the ration daily from Day 0 to 90. Behavioural evaluation, scrotal circumference measurement, testicular ultrasonography and blood sampling for serum testosterone analysis were performed on Days 0, 15, 30, 45, 60, 75 and 90.

The IC group received two subcutaneous injections of 1 mL of Bopriva (400 μg GnRH‐protein conjugate; Zoetis, Australia) administered in the neck region on Day 0 and 21. The PFA group received 50 g/day/animal of a commercial phytogenic feed additive (Stop‐Hop Premix; İdeal, İzmir, Türkiye) throughout the 90‐day experimental period. Throughout the study, all bulls were managed by the same farm personnel under identical husbandry conditions. The overall experimental workflow, including treatment allocation, intervention schedule and follow‐up assessments, is illustrated in Figure 1.

All clinical, reproductive, ultrasonographic and hormonal evaluations were performed at baseline (Day 0) and repeated on Days 15, 30, 45, 60, 75 and 90 during the experimental period. The study was terminated on Day 90 immediately after the final assessments; therefore, no post‐treatment follow‐up period was included and the persistence or reversibility of treatment effects beyond the experimental period was not evaluated.

### 2.2 Management and Nutrition

Throughout the experimental period, bulls were housed in separate group pens corresponding to their treatment groups, under identical environmental and management conditions. Animals had ad libitum access to fresh water and wheat straw as the primary roughage source. Each bull received 4 kg/day of commercial concentrate feed, divided into two equal meals. On a dry‐matter basis, the concentrate ration contained 14% crude protein, 7.3% crude fibre, 3.1% crude fat and 7.5% crude ash. Feeding management remained consistent throughout the study to minimize nutritional variability between groups. A phytogenic feed additive was incorporated into the basal diet and administered daily throughout the 90‐day experimental period. The phytogenic supplement was supplied as a powdered premix and top‐dressed individually onto the concentrate ration once daily during morning feeding. The phytogenic feed additive used in this study was a commercially available premix (Stop‐Hop, Türkiye) in powder form. According to the manufacturer's declaration, each kilogram of the product contained 5.000 mg of fennel essential oil, 5.000 mg of garlic essential oil, 3.500 mg of thyme essential oil, 3.500 mg of rosemary essential oil, 1.200 mg of peppermint essential oil and 982.3 g of aluminosilicate as the carrier material. The calculated daily amount was thoroughly mixed with the concentrate ration immediately before feeding and offered once daily. Feed distribution was performed at the same time each day by the same stockperson throughout the experimental period to ensure consistency in feed preparation and administration. No refusals associated with the phytogenic supplement were observed during the study period. The PFA group received 50 g/day/animal of a commercial phytogenic feed additive throughout the 90‐day experimental period. Animals were housed in group pens and fed from group feeders under identical management conditions. During feeding, animal caretakers visually monitored feed intake to ensure that the concentrate ration containing the phytogenic premix was consumed under routine farm conditions.

### 2.3 Assessment of Behavioural Observations

Behavioural activity was evaluated throughout the 90‐day experimental period using standardized direct visual observations performed by the same experienced observer, who was familiar with the animals and blinded to the study hypotheses. Behavioural assessments were conducted twice daily between 08:00–09:00 and 16:00–17:00 h. To minimize observer‐induced stress, the bulls were monitored within their routine housing environment. Observations specifically targeted general activity concentrations, indicators of restlessness and displays of agonistic or aggressive behaviours (e.g., head‐butting, pawing). These findings were recorded as qualitative observations to provide supplementary ethological context regarding the animals' overall well‐being and adaptation to the treatments throughout the 90‐day study.

### 2.4 Measurement of Scrotal Circumference

Scrotal circumference (SC) was measured in all bulls before treatment initiation (Day 0) and subsequently on Days 15, 30, 45, 60, 75 and 90 throughout the experimental period. All measurements were performed by the same experienced investigator to minimize inter‐observer variation. Scrotal circumference (SC) was measured to assess testicular size. Before measurement, the testes were gently pushed into the distal portion of the scrotum and stabilized by placing the thumb and fingers on the lateral aspects of the scrotum to ensure they were positioned side‐by‐side. The SC was then determined at the maximum testicular diameter using a flexible, non‐stretchable measuring tape and the results were recorded in centimetres (cm) and identical measurement procedures were applied at each evaluation time.

### 2.5 Determination of Serum Testosterone Concentrations

Blood samples were collected from the jugular vein before treatment initiation (Day 0) and on Days 15, 30, 45, 60, 75 and 90, immediately before routine handling procedures between 13:00 and 15:00 h to account for diurnal variation in serum testosterone concentrations. Samples were allowed to clot at room temperature, then centrifuged at 1200×*g* for 12 min. The separated serum was stored at −20°C until analysis. Serum testosterone concentrations were quantified in duplicate using a commercial bovine‐specific ELISA kit (Cat. No. EA0019BO, BT LAB, Shanghai, China) in accordance with the manufacturer's protocols. According to the manufacturer, the assay exhibits an inter‐assay coefficient of variation (CV) < 10%, with low intra‐assay variability as reported in the kit validation data. The kit had a sensitivity of 0.051 ng/mL and a detection range of 0.1–40 ng/mL. Absorbance was measured using an ELISA microplate reader. The ELISA method was selected for its practicality, accessibility and widespread use in veterinary reproductive studies involving bovine serum testosterone measurements.

### 2.6 Ultrasonographic Evaluation of Testicular Parenchyma

**FIGURE 2:**
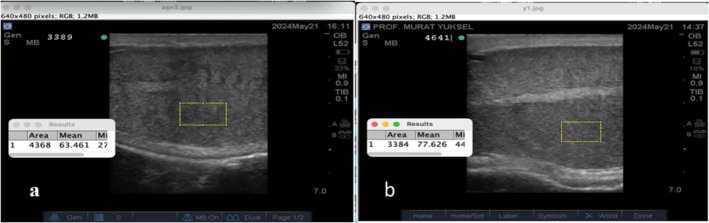
Representative B‐mode ultrasonograms illustrating the quantitative echotexture analysis of the bovine testicular parenchyma. Measurements were performed on longitudinal images of the right testis in (a) the immunocastration group and (b) the phytogenic feed additive group. Yellow rectangles indicate the Regions of Interest (ROI) selected within the testicular parenchyma for image analysis. The inset tables show the corresponding Mean Grey Value (MGV) measurements calculated from each ROI and used for subsequent statistical analyses.

Ultrasonographic examinations were performed before treatment initiation (Day 0) and repeated on Days 15, 30, 45, 60, 75 and 90. Ultrasonographic examinations were performed using a B‐mode real‐time ultrasound system equipped with a 7 MHz linear transducer (Fujifilm Sonosite M‐Turbo Portable Ultrasound, Bothell, WA, USA). All system settings, including gain, focus and depth, were kept constant throughout the study to ensure consistent quantitative analysis. The testicular parenchyma was evaluated in both longitudinal and transverse planes. Echogenicity assessment was performed on representative longitudinal scans of the right testis (Figure 2). Quantitative echotexture analysis was conducted using ImageJ software (National Institutes of Health, Bethesda, MD, USA). For each ultrasonographic image, three rectangular regions of interest (ROI; approximately 1 × 1 cm) were selected within homogeneous areas of the testicular parenchyma while carefully avoiding the mediastinum testis and major vascular structures. The Mean Grey Value (MGV), representing the average pixel intensity within these ROIs, was automatically calculated by the software and used as an objective indicator of testicular echogenicity. Lower MGV values represented a more hypoechoic testicular parenchyma (darker) ultrasonographic appearance, whereas higher MGV values reflected increased echogenicity.

### 2.7 Statistical Analysis

Statistical analyses were performed using IBM SPSS Statistics (Version 27.0; IBM Corp., Armonk, NY, USA). Continuous variables are presented as means ± standard deviations (Means ± SD), unless otherwise stated. Baseline differences between treatment groups (Day 0) were evaluated before longitudinal analyses. Repeated measurements of scrotal circumference, serum testosterone concentration and testicular echogenicity were analysed using linear mixed models (LMMs). Treatment group, time and their interaction were included as fixed effects, while animal ID was included as a random effect to account for repeated measurements from the same individual. Parameters were estimated using Restricted Maximum Likelihood (REML), and a first‐order autoregressive [AR(1)] covariance structure was applied to model within‐animal correlations over time. Model assumptions were assessed by visual inspection of residual plots and evaluation of residual normality. When significant main effects or interactions were detected, Bonferroni‐adjusted pairwise comparisons of the estimated marginal means (EMMeans) were performed both between treatment groups at each time point and within treatment groups across time. Pearson correlation analysis was performed to evaluate the relationships among serum testosterone concentrations, scrotal circumference and mean grey value (MGV). Statistical significance was accepted at *p* < 0.05. For descriptive purposes, coefficients of variation (CVs, %) were calculated for serum testosterone concentration, scrotal circumference and mean grey value to quantify the biological variability of repeated measurements throughout the experimental period.

## 3 Results

### 3.1 Behavioural Observations

No systemic adverse reactions or clinically abnormal findings were observed in either the immunocastration (IC) or phytogenic feed additive (PFA) group throughout the 90‐day experimental period. Mild reductions in sexual behaviour and temporary restlessness were observed in both groups during the first week following treatment initiation based on daily clinical observations. These behavioural changes gradually diminished over time, and no persistent behavioural abnormalities were recorded during the remaining observation period. By Day 15, behavioural activity had stabilized in both groups, characterized by reduced mounting attempts and decreased vocalization and all animals remained in good general health throughout the study. For the remainder of the experimental period, subjects in both groups maintained their baseline temperaments. Notably, no instances of increased aggressiveness, loss of appetite, or local injection site reactions (in the IC group) were observed, indicating that both Bopriva treatment and Stop‐Hop supplementation were well‐tolerated in young Holstein bulls.

### 3.2 Scrotal Circumference

Scrotal circumference measurements recorded throughout the experimental period are presented in Table 1. Baseline scrotal circumference did not differ significantly between the IC and PFA groups (*p* = 0.28). From Day 15 onward, scrotal circumference was significantly lower in the IC group than in the PFA group at each evaluation time point (*p* < 0.05). Linear mixed model analysis revealed significant effects of treatment group (*F* = 6.433, *p* = 0.027, *η* ^2^ = 0.095), time (*F* = 7.259, *p* < 0.001, *η* ^2^ = 0.414) and the treatment × time interaction (*F* = 3.942, *p* = 0.002, *η* ^2^ = 0.277) on scrotal circumference. Throughout the experimental period, scrotal circumference decreased in both groups, with a substantially greater reduction observed in the immunocastration (IC) group than in the phytogenic feed additive (PFA) group. Specifically, the IC group exhibited a progressive and marked reduction in SC, concluding the study at 20.92 cm, whereas the PFA group maintained a relatively stable course, finishing at 26.79 cm. Bonferroni‐adjusted pairwise comparisons showed that the significant within‐group reductions in scrotal circumference occurred exclusively in the immunocastration group, whereas no significant temporal changes were detected in the phytogenic feed additive group. Bonferroni‐adjusted pairwise comparisons indicated that significant within‐group reductions in scrotal circumference occurred only in the immunocastration group, whereas no significant temporal changes were observed in the phytogenic feed additive group. Estimated marginal means (EMMeans) derived from the fitted Linear Mixed Model are presented throughout the Results section. Unlike simple arithmetic means, EMMeans are model‐adjusted estimates that account for the correlation among repeated measurements obtained from the same animals over time. Across all repeated observations, the coefficients of variation were 32.3% for serum testosterone concentration, 16.6% for scrotal circumference and 4.4% for mean grey value (MGV). These findings indicate substantial biological variability in testosterone concentrations, moderate variability in scrotal circumference and high repeatability of ultrasonographic echogenicity measurements throughout the experimental period.

**TABLE 1:** Effects of immunocastration and phytogenic feed additive on scrotal circumference (cm) in young bulls during the 90‐day experimental period.

Time (Days)	IC group (*n* = 6)	PFA group (*n* = 7)	*p* ^a^	Source of variation	*F*	*p*	*η* ^2^
0	26.08 ± 4.60	28.50 ± 2.81	0.28				
15	23.58 ± 4.12	28.29 ± 2.78	0.03				
30	23.25 ± 3.91	27.93 ± 3.30	0.02	Group	6.433	0.027	0.095
45	22.58 ± 4.35	28.07 ± 3.07	0.01	Time	7.259	< 0.001	0.414
60	23.00 ± 3.83	27.71 ± 3.07	0.02	Group × Time	3.942	0.002	0.277
75	21.25 ± 4.01	27.15 ± 2.80	0.01				
90	20.92 ± 3.93	26.79 ± 3.03	0.01				

*Note:* Values are presented as estimated marginal means (EMMeans ± SE) obtained from the fitted linear mixed model after adjustment for repeated measurements. Pairwise comparisons between treatment groups at each time point and within‐group comparisons across time were performed using Bonferroni‐adjusted post hoc tests. *F*, *p* and partial *η* ^2^ values represent the fixed effects estimated from the Linear Mixed Model.

^a^ Represents the *p*‐value for inter‐group comparison at each time point. Animal ID was included as a random effect in the Linear Mixed Model.

### 3.3 Serum Testosterone Concentrations

Serum testosterone concentrations measured throughout the experimental period are presented in Table 2. Baseline testosterone concentrations were similar between the IC and PFA groups (*p* > 0.05). Testosterone concentrations in the IC group decreased significantly after treatment initiation (decreasing from 9.37 ng/mL on Day 0 to 5.63 ng/mL by Day 15 and subsequently stabilizing between 4.0 and 4.6 ng/mL for the remainder of the study) and remained lower than those of the PFA group throughout the subsequent sampling periods (*p* < 0.05). In contrast, testosterone concentrations in the PFA group showed only minor fluctuations throughout the experimental period, ranging from 8.9 to 9.3 ng/mL. Linear mixed model analysis revealed significant effects of treatment group, time and the treatment × time interaction on serum testosterone concentrations (Table 2). Bonferroni‐adjusted pairwise comparisons indicated significant decreases in serum testosterone concentrations from Day 15 onwards in the immunocastration group; no significant within‐group differences were observed in the phytogenic feed additive group.

**TABLE 2:** Effects of immunocastration and phytogenic feed additive on serum testosterone concentrations (ng/mL) in young bulls during the 90‐day experimental period.

Time (Days)	IC group (*n* = 6)	PFA group (*n* = 7)	*p* ^a^	Source of variation	*F*	*p*	*η* ^2^
0	9.37 ± 0.66	9.34 ± 0.59	0.93				
15	5.63 ± 0.60	9.23 ± 0.58	< 0.001				
30	4.41 ± 0.50	9.25 ± 0.67	< 0.001	Group	483.72	< 0.001	0.90
45	4.11 ± 0.12	9.16 ± 0.66	< 0.001	Time	42.59	< 0.001	0.55
60	4.04 ± 0.10	8.97 ± 0.75	< 0.001	Group × Time	36.05	< 0.001	0.51
75	4.56 ± 0.47	8.89 ± 0.49	< 0.001				
90	4.36 ± 0.42	9.01 ± 0.62	< 0.001				

*Note:* Values are presented as estimated marginal means (EMMeans ± SE) obtained from the fitted linear mixed model after adjustment for repeated measurements. Pairwise comparisons between treatment groups at each time point and within‐group comparisons across time were performed using Bonferroni‐adjusted post hoc tests. *F*, *p* and partial *η* ^***2***^ values represent the fixed effects estimated from the Linear Mixed Model.

^a^ Represents the *p*‐value for inter‐group comparison at each time point. Animal ID was included as a random effect in the Linear Mixed Model.

### 3.4 Testicular Echogenicity

Testicular ultrasonographic measurements obtained during the experimental period are summarized in Table 3. Mean grey value changed over time in both groups. Significant treatment × time interactions were detected for the evaluated ultrasonographic variables (*p* < 0.05). Compared with the PFA group, the IC group exhibited a greater reduction in testicular dimensions and mean grey value during the experimental period. At the initiation of the study (Day 0), no significant difference was observed between the groups (*p* = 0.90), confirming baseline homogeneity in the parenchymal appearance. However, a significant divergence between the treatments began to manifest from Day 30 onwards (*p < 0.05*) and persisted throughout the remainder of the 90 days. Mean grey values decreased over time in the IC group (from 65.08 ± 0.57 on Day 0 to 58.82 ± 2.16 by Day 90), whereas only minor fluctuations were observed in the PFA group (ranging between 64.86 ± 2.68 and 65.76 ± 1.64). Bonferroni‐adjusted pairwise comparisons demonstrated that the reduction in mean grey value became significant from Day 30 onwards in the immunocastration group; no significant temporal changes were observed in the phytogenic feed additive group.

**TABLE 3:** Effects of immunocastration and phytogenic feed additive on testicular echogenicity (mean pixel intensity) in young bulls during the 90‐day experimental period.

Time (Days)	IC group (*n* = 6)	PFA group (*n* = 7)	*p* ^a^	Source of variation	*F*	*p*	*η*2
0	65.08 ± 0.57	64.96 ± 0.43	0.90				
15	64.18 ± 0.95	65.35 ± 0.98	0.052				
30	63.74 ± 0.82	65.29 ± 1.44	**< 0.05**	Group	17.021	0.001	0.57
45	61.86 ± 1.48	65.76 ± 1.64	**< 0.01**	Time	5.657	< 0.001	0.36
60	60.66 ± 1.17	65.24 ± 2.48	**< 0.01**	Group × Time	6.121	< 0.001	0.37
75	59.45 ± 2.02	65.24 ± 2.82	**< 0.01**				
90	58.82 ± 2.16	64.86 ± 2.68	**< 0.01**				

*Note:* Values are presented as estimated marginal means (EMMeans ± SE) obtained from the fitted linear mixed model after adjustment for repeated measurements. Pairwise comparisons between treatment groups at each time point and within‐group comparisons across time were performed using Bonferroni‐adjusted post hoc tests. *F*, *p* and partial *η* ^2^ values represent the fixed effects estimated from the Linear Mixed Model. Statistically significant differences are indicated by *p* < 0.05.

^a^ Represents the *p*‐value for inter‐group comparison at each time point. Animal ID was included as a random effect in the Linear Mixed Model.

### 3.5 Correlation Analysis

Pearson correlation analysis further demonstrated significant positive associations among serum testosterone concentrations, scrotal circumference and testicular echogenicity (Table 4). Serum testosterone concentrations showed a moderate positive correlation with scrotal circumference (*r* = 0.555, *p* < 0.001) and a strong positive correlation with mean grey value (*r* = 0.637, *p* < 0.001). Likewise, scrotal circumference was positively correlated with mean grey value (*r* = 0.591, *p* < 0.001). These findings support the close association between endocrine activity, morphometric measurements and quantitative ultrasonographic characteristics throughout the experimental period.

**TABLE 4:** Pearson correlation coefficients among serum testosterone concentration, scrotal circumference and testicular echogenicity (mean grey value) in young Holstein bulls during the experimental period.

Variables	Pearson's *r*	*p*
Testosterone vs. SC	0.555	< 0.001
Testosterone vs. MGV	0.637	< 0.001
SC vs. MGV	0.591	< 0.001

*Note:* Pearson correlation coefficients (*r*) were used to assess the linear relationships among serum testosterone concentration, scrotal circumference and mean grey value (MGV). Correlation analysis was performed using pooled repeated observations from all animals throughout the experimental period (*n* = 91). Statistical significance was set at *p <* 0.05.

## 4 Discussion

The present study compared the effects of immunocastration and a phytogenic feed additive on behavioural observations, serum testosterone concentrations, scrotal circumference and quantitative ultrasonographic characteristics of the testes in young Holstein bulls. Distinct treatment responses were observed throughout the 90‐day experimental period. Immunocastration was associated with marked reductions in serum testosterone concentrations, scrotal circumference and testicular echogenicity, whereas bulls receiving the phytogenic feed additive maintained relatively stable reproductive measurements. To our knowledge, few studies have simultaneously evaluated the temporal relationships among endocrine, morphometric and quantitative ultrasonographic changes following immunocastration in bulls. The behavioural quiescence observed in the PFA group, occurring without apparent HPG‐axis suppression, might be associated with the specific neurophysiological properties of the essential oil blend. Substantial evidence regarding the neurophysiological impact of essential oils is well‐documented, with recent studies highlighting their capacity to significantly modulate the sympathetic nervous system and various neurotransmitter systems (Lizarraga‐Valderrama 2020; Soares et al. 2022). These phytogenic compounds, including those derived from aromatic herbs, are recognized for their bioactive potential in providing neuroprotective and regulatory effects through multi‐target pharmacological responses. As detailed by Lizarraga‐Valderrama (2020), essential oils can interact with key neurotransmitter pathways—including serotonergic, dopaminergic and GABAergic systems—potentially translating into measurable brain activity changes without impairing motor coordination. This dual action on the hypothalamic–pituitary–adrenal (HPA) axis and the sympathetic nervous system could promote a state of relaxation and reduce reactivity to environmental stimuli. Specifically, these bioactive substances may foster sedation by increasing serotonin concentrations and concurrently decreasing glucocorticoid concentrations, thereby stabilizing the animals' physiological response to stressors (Zhang et al. 2016). Although the precise mechanism remains unclear, the available literature suggests that essential oils may influence behavioural responses through modulation of neurotransmitter systems and stress‐related neuroendocrine pathways. This mechanism could explain the behavioural observations recorded in the present study without requiring suppression of the hypothalamic–pituitary–gonadal axis. The relatively stable reproductive measurements observed in the PFA group suggest that supplementation with the evaluated phytogenic formulation did not induce measurable adverse effects on the endocrine or ultrasonographic variables assessed during the experimental period. In line with this, Wells (2024) asserts that essential oils can provide beneficial biological effects in ruminants without inducing adverse impacts, suggesting that they should be considered a significant component of animal health programs. Managing aggressive behaviour in young bulls typically necessitates a trade‐off between handling ease and the anabolic benefits of testosterone. Under the conditions of the present study, bulls receiving the phytogenic feed additive (PFA) maintained relatively stable reproductive measurements while exhibiting behavioural changes comparable to those observed following immunocastration.

The rapid decline in serum testosterone concentrations observed after immunocastration was accompanied by a progressive reduction in scrotal circumference, supporting the expected endocrine response following GnRH immunization. While both groups exhibited sustained calmness throughout the 90 days, a clear divergence emerged in reproductive parameters; the IC group underwent significant testicular regression and hormonal decline, whereas the PFA group maintained relatively stable reproductive parameters. Unlike anti‐GnRH, supplementation with the evaluated phytogenic formulation was not associated with marked reductions in serum testosterone concentrations, scrotal circumference, or testicular echogenicity during the experimental period (D'Occhio et al. 2001; Janett et al. 2012a). The significant group–by–time interaction (*p* < 0.001) observed for both testis size (Table 1) and serum testosterone (Table 2) underscores the rapid and sustained efficacy of immunocastration starting from Day 15. The initial reduction in these parameters in the IC group aligns with previous observations regarding the acute effects of GnRH immunization. Furthermore, the early onset of testosterone suppression observed in our study is consistent with Theubet et al. (2010), who reported a similarly swift hormonal blockade in peripubertal bulls shortly after the booster dose. However, a notable divergence was observed regarding both the onset of suppression and the recovery phase compared to other studies. While D'Occhio et al. (2001) reported that 65% of 22‐month‐old bulls re‐initiated testicular growth only 2 months after treatment, our results in the IC group showed no such rebound. Similarly, Bolado‐Sarabia et al. (2018) observed that testosterone concentrations in Holstein bulls remained comparable to those of intact animals until Day 101, with significant suppression becoming evident only after Day 181. This discrepancy suggests that the efficacy and timing of the immune response are significantly influenced by the functional status of the hypothalamic–pituitary‐gonadal axis at the time of primary immunization. In our study, the older age of the bulls (12–15 months) likely coincided with a more active GnRH secretion profile, making the antibody‐mediated blockade more immediately apparent and robust compared to the younger, prepubertal subjects (7–8 months) used by Bolado‐Sarabia et al. (2018). Furthermore, the onset of suppression in our study occurred earlier than in mature bulls; while Doroteu et al. (2021) reported significant reductions in testosterone and scrotal circumference in 24‐month‐old Nelore bulls only after Day 60, our Holstein bulls exhibited these changes as early as Day 15. Throughout the 90 days, testosterone and scrotal circumference in the IC group remained consistently suppressed. This observed stability and uniformity of the response further support that the age at first vaccination may represent an important determinant for the duration of GnRH neutralization (Janett et al. 2012a; Amatayakul‐Chantler et al. 2012). In support of this, consistent with our findings, Xu et al. (2018) demonstrated that active immunization of 13‐month‐old Holstein bulls resulted in a sharp decrease in testosterone concentrations and a significant arrest of testicular growth.

**FIGURE 3:**
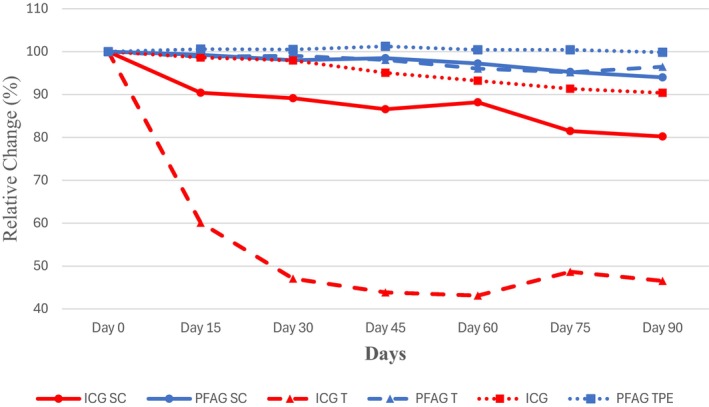
Relative changes in serum testosterone, scrotal circumference and testicular parenchymal echogenicity in the immunocastration and phytogenic feed additive groups during the 90‐day experimental period. Abbreviations: ICG, immunocastration group; PFAG, phytogenic feed additive group; T, testosterone; SC, scrotal circumference; TPE, testicular parenchymal echogenicity.

In the present study, the progressive reduction in testicular parenchymal echogenicity (TPE) closely followed the sharp decline in serum testosterone concentrations observed during the first 30 days in the ICG (Figure 3). This temporal decrease in mean grey value may reflect progressive changes in testicular parenchymal echogenicity associated with androgen deprivation. However, because histopathological evaluation was not performed, these ultrasonographic findings should be interpreted as indirect indicators rather than direct evidence of structural tissue alterations. Consistent with Abecia et al. (2020), who reported a direct relationship between serum testosterone concentrations and testicular echotexture in rams, our findings indicate that the loss of androgenic support leads to a decrease in overall testicular echotexture. This reduction is likely attributed to the collapse of seminiferous tubule lumens and the subsequent decrease in intratesticular fluid volume, which mirrors the functional inactivation of the Leydig cells following GnRH immunization (Doroteu et al. 2021). Quantitative digital analysis of testicular ultrasonograms (Table 3) enabled objective, non‐invasive monitoring of temporal changes in testicular parenchymal echogenicity throughout the experimental period. In our study, a highly significant group‐by‐time interaction (*p* < 0.001) was observed for testicular echotexture, with the IC group showing a distinct divergence starting from Day 30 and persisting until Day 90. This temporal shift in MGV reflects the progressive physiological regression of the seminiferous tubules and the suppression of spermatogenesis following GnRH immunization. Supporting this relationship, Montes‐Garrido et al. (2022) demonstrated in rams that a reduction in the density of hypoechogenic areas is significantly associated with a decrease in tubular area and diameter. In our study, the observed decline in MGV values from day 30 onwards in the IC group indicates a transition toward a darker (more hypoechoic) testicular parenchyma, consistent with progressive changes in testicular echotexture. These findings align with Ulker et al. (2005), who reported a similar transition in the testicular parenchyma of rams following immunocastration, suggesting a common response pattern in ruminant testicular tissue to hormonal deprivation. Furthermore, these quantitative shifts are strongly supported by findings in bulls; Brito et al. (2012) reported that testicular pixel intensity in bulls is significantly correlated with seminiferous tubule diameter and seminiferous epithelium area. Collectively, these findings support the usefulness of quantitative ultrasonographic image analysis as a non‐invasive method for monitoring temporal changes in testicular echogenicity following immunocastration. The observed reduction in mean grey value in the immunocastration group is consistent with previous reports describing ultrasonographic changes following suppression of testicular activity. However, because histopathological evaluation was not performed, these image‐derived findings should be interpreted as indirect indicators of alterations in testicular parenchymal characteristics rather than direct evidence of tissue structural or functional changes. The significant main effects of Group (*p* < 0.001) and Time (*p* < 0.001) further underscore the dynamic nature of these parenchymal alterations. While the IC group underwent a structural shift potentially associated with hormonal deprivation, the observed echotextural stability in the PFA group suggests that this phytogenic approach may not adversely affect testicular tissue composition under the conditions of this study. This indicates that the behavioural modulation achieved in the PFA group appears to occur without the degree of parenchymal regression observed in the IC group, thereby supporting the maintenance of the ultrasonographic echotexture. The relatively stable ultrasonographic findings observed in the PFA group indicate that supplementation with the evaluated phytogenic formulation was not associated with measurable alterations in testicular echogenicity during the experimental period. From a practical perspective, the present findings indicate that the evaluated phytogenic feed additive may represent a potential management strategy for reducing behavioural reactivity without producing measurable changes in the reproductive variables evaluated during the study period. However, because semen quality and fertility were not assessed, no conclusions can be drawn regarding its effects on reproductive performance.

The findings of the present study should be interpreted in light of its experimental limitations. Animals were monitored only during the 90‐day treatment period; therefore, the persistence or reversibility of the observed endocrine and ultrasonographic changes following treatment cessation remains unknown. In addition, semen quality, fertility and histopathological evaluations were beyond the scope of the present study. Future studies incorporating post‐treatment follow‐up, semen quality evaluation, fertility assessment and histopathological examination are warranted to further clarify the long‐term reproductive effects of both immunocastration and phytogenic feed additive supplementation.

## 5 Conclusions

In conclusion, the present findings indicate that both immunocastration and phytogenic feed additive supplementation may represent useful approaches for managing aggressive behaviour in young Holstein bulls, although they appear to act through distinct biological pathways. While active immunization against GnRH was associated with sustained suppression of serum testosterone concentrations and changes in scrotal circumference and testicular echogenicity, supplementation with the phytogenic feed additive was associated with relatively stable serum testosterone concentrations and ultrasonographic testicular characteristics while exhibiting behavioural observations comparable to those recorded in the immunocastration group during the study period. These findings suggest that such non‐hormonal interventions may provide an alternative approach for improving animal handling under intensive beef production systems. Future research should evaluate these findings in larger animal populations and investigate their long‐term effects on carcass characteristics, meat quality, semen quality, fertility and post‐treatment reversibility. Furthermore, studies investigating the neurobiological mechanisms underlying the effects of essential oil blends would contribute to a better understanding of non‐hormonal behavioural modulation. However, the absence of an untreated control group, a quantitative behavioural scoring system and post‐treatment follow‐up should be considered when interpreting the present findings.

## Author Contributions

N.C.: conceptualization, supervision and writing – review and editing. F.K.: investigation, project administration, data curation and writing – original draft preparation; O.K.Y.: Methodology and validation. M.Y.: methodology and resources. H.B.D.: methodology. C.T.A.: methodology, formal analysis and visualization; All authors have read and agreed to the published version of the manuscript.

## Funding

This study was supported by the Hatay Mustafa Kemal University Scientific Research Projects Coordination Office (Antakya, Türkiye) under project number 24.GAP.012.

## Conflicts of Interest

The authors declare no conflicts of interest.

## Data Availability

The datasets generated and analysed during the current study are available from the corresponding author on reasonable request.
